# Tree-Level Growth Patterns and Genetic Associations Depict Drought Legacies in the Relict Forests of *Abies marocana*

**DOI:** 10.3390/plants12040873

**Published:** 2023-02-15

**Authors:** Belén Méndez-Cea, Isabel García-García, Raúl Sánchez-Salguero, Víctor Lechuga, Francisco Javier Gallego, Juan C. Linares

**Affiliations:** 1 Departamento de Genética, Fisiología y Microbiología, Unidad de Genética, Facultad de CC Biológicas, Universidad Complutense de Madrid, 28040 Madrid, Spain; 2 Departamento de Sistemas Físicos, Químicos y Naturales, Universidad Pablo de Olavide, 41013 Sevilla, Spain; 3 Centro de Estudios Avanzados en Ciencias de la Tierra, Energía y Medio Ambiente (CEACTEMA), Universidad de Jaén, 23071 Jaén, Spain

**Keywords:** *Abies marocana*, drought sensitivity, tree age, selection signature, genotype–phenotype associations, dendrochronology

## Abstract

The frequency and intensity of drought events are increasing worldwide, challenging the adaptive capacity of several tree species. Here, we evaluate tree growth patterns and climate sensitivity to precipitation, temperature, and drought in the relict Moroccan fir *Abies marocana*. We selected two study sites, formerly stated as harboring contrasting *A. marocana* taxa (*A. marocana* and *A. tazaotana*, respectively). For each tree, dendrochronological methods were applied to quantify growth patterns and climate–growth sensitivity. Further, ddRAD-seq was performed on the same trees and close saplings to obtain single nucleotide polymorphisms (SNPs) and related genotype–phenotype associations. Genetic differentiation between the two studied remnant populations of *A. marocana* was weak. Growth patterns and climate–growth relationships were almost similar at the two sites studied, supporting a negative effect of warming. Growth trends and tree size showed associations with SNPs, although there were no relationships with phenotypes related to climatic sensitivity. We found significant differences in the SNPs subjected to selection in the saplings compared to the old trees, suggesting that relict tree populations might be subjected to genetic differentiation and local adaptation to climate dryness. Our results illustrate the potential of tree rings and genome-wide analysis to improve our understanding of the adaptive capacity of drought-sensitive forests to cope with ongoing climate change.

## 1. Introduction

Trees are sessile organisms with a long generation time, which makes them vulnerable to climate change and hindered by fast adaptations [1,2,3]. The frequency and intensity of drought events are increasing worldwide, triggering extensive dieback and mortality in many forest ecosystems [4,5,6]. At a regional scale, the Mediterranean basin is highly vulnerable to the ongoing warming, a trend that is predicted to worsen [7]. As a consequence, drought-sensitive forests may be increasingly stressed, as water shortage impairs the functioning of trees by reducing their photosynthesis and growth rates [8,9]. The circum-Mediterranean firs can be considered one of the most sensitive tree species to climate change [10,11]. This taxonomic group is subdivided into two sections: *Abies* and *Piceaster* [12]. It is remarkable that the *Piceaster* group is formed by the most ancient lineages, currently relict tree species, namely: *Abies pinsapo* Boiss., *Abies marocana* Trab., *Abies tazaotana* Villar, and *Abies numidica* Carr [12]. These species are drought-sensitive, although they have several physiological mechanisms which allow them to cope with water stress, such as an early stomatal closure [13]. Furthermore, relict tree species rely on mechanisms promoting population persistence, which raises several uncertainties concerning their future prospects [14]. Relict populations persist geographically isolated in enclaves of benign environmental conditions within a regional climate significantly hotter and dryer than those tolerated by the species [11]. Hence, complex ecological and evolutionary factors determine population dynamics, and point out the need for a proper understanding of the key mechanisms implied in population persistence [14].

In this work, two remnant forests of the Moroccan fir (*A. marocana*) were investigated. Recent studies reported increasing threats induced by drought stress in the relative *A. pinsapo* in south Spain [11]. Our knowledge about drought resilience in *A. marocana* forests is still limited, while useful studies about tree growth and stand dynamics have been recently performed [15,16]. On the other hand, previous genetic studies focused on *A. marocana* used molecular markers, such as microsatellites based on single sequence repeats (SSRs), mainly mitochondrial or chloroplast SSR, and single nucleotide polymorphisms (SNPs), aimed to determine genetic diversity and populations’ genetic structure [17,18,19,20].

The genetic knowledge about Moroccan fir species is still scarce. One of the main reasons is the large size of the conifer genomes and the high number of repetitive sequences [3]. In addition, the absence of reference genomes for most tree species makes these genetic studies challenging [3]. Nevertheless, in recent times, the advances in next-generation sequencing (NGS) offer a wide range of possibilities to perform studies with giga-genomes, even without a reference. One of these techniques is genotyping by sequencing (GBS) using double digest restriction-site-associated DNA (ddRAD-seq) [21]. This technique provides new molecular markers and increases the genetic knowledge of non-model species. In addition, the molecular markers obtained with this technique allow us to carry out association studies between single nucleotide polymorphisms (SNPs) and local adaptations to the environment [22].

Despite the above-mentioned advances in genotyping techniques, our knowledge of the potential for evolutionary responses to climate change is very limited for most tree species [3,23]. Indeed, there is still no agreement on the relationship between individual genetic diversity and tree growth, with positive relationships [24,25,26] or no associations [27,28]. Recently, the combination of genetic and dendroecological data has been proposed as a reliable framework to obtain a new class of phenotypes (dendrophenotypes) for genetic associations [29,30,31,32,33]. Through the investigation of tree rings, dendroecology allows the quantification of climatic constraints that are exerted on trees [11]. Hence, the analysis of the relationships between growth and climate variables enables us to assess tree-level sensitivity to climatic constraints, reflecting individual growth-limiting factors [31]. Here, such tree-ring characteristics reflecting climate sensitivity have been used as phenotypic traits in the context of quantitative genetics. Thus, we performed genotype-(dendro)phenotype associations (GPA) to identify genetic regions involved in tree growth patterns and their responses to climate stress [31].

Our specific aims are (i) to quantify local climate, genetic diversity, and secondary growth trends of the relict tree *A. marocana*, considering two nuclei potentially harboring genetic differentiation; (ii) to quantify tree-level climate sensitivity as a phenotypic trait; (iii) to quantify the genetic structure of such contrasting sites and between old trees and saplings, in order to account for spatial (between sites) and temporal (between cohorts) shifts in genetic diversity; (iv) to perform genotype-(dendro)phenotype associations to identify genetic regions involved in long-term *A. marocana* growth patterns and its response to climate stress at an individual tree level.

## 2. Results

### 2.1. Climate and Dendrochronology Studies

Total annual precipitation was not reduced in the study area over the 20th century; indeed, the trend obtained for the period of 1921–2020 was significantly positive (Figure S1a; Table 1), mainly due to a positive trend in winter precipitation. The analysis of the period 1961–2020 depicts contrasting trends in the intra-annual patterns of precipitations, with summer precipitation showing a significant decline, while the rest of the seasons showed no significant changes (Table 1). On the contrary, trends in mean temperature were consistently positive, supporting a warming of the study area (Figure S1a; Table 1), mainly during winter and spring, supporting an estimation of about 1.7 °C mean annual temperature rise between 1961 and 2020.

**Figure 1:**
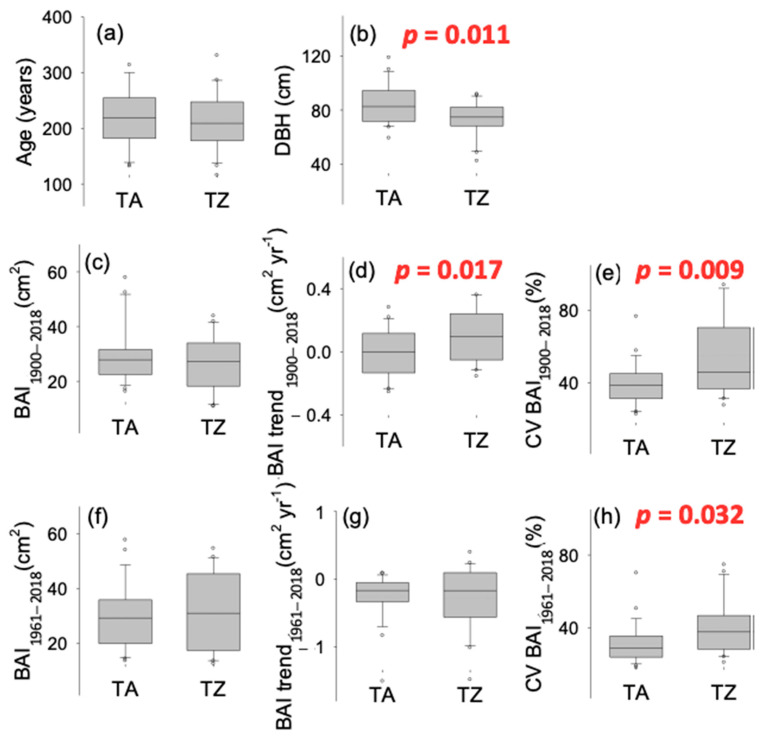
Comparison of tree-scale growth patterns estimated as the basal area increments (BAI) of tree rings obtained in *Abies marocana* for Talassemtane (TA) and Tazaot (TZ). Tree age (**a**) was estimated at coring height, according to tree-ring dating. DBH (**b**) indicates the tree diameter at 1.3 m from the ground. Mean growth was computed comparing the periods 1900–2018 (**c**) and 1961–2018 (**f**). BAI trend (**d**,**g**) was estimated as the slope of BAI against the time. CV (**e**,**h**) indicates the coefficient of variation, estimated as (100*) the ratio of the BAI standard deviation divided by the mean. Significant *p*-values for ANOVA are indicated.

Extreme drought events (estimated as SPEI values below −1.5; Figure S1b) increased in frequency toward the last third of the 20th century and the onset of the 21st. Furthermore, summer SPEI shows negative values for almost two decades at the onset of the 21st century. Hence, except that of 1945, all the extreme drought events registered in the dataset took place after 1983, and six drought events occurred in a period of roughly two decades, between 1995 and 2016 (1995, 1999, 2005, 2012, 2014, 2016; Figure S1b). The growth pattern (BAI time series) was very similar between TA and TZ sites (r = 0.66 for the period 1900–2018, *p* < 0.01; r = 0.78 for the period 1961–2018, *p* < 0.01; Figure 1 and Figure S2). *A. marocana* trees were sampled at similar elevation (around 1700 m, Table 2) and old trees showed similar age in both study sites (about 220 years old; Table 2, Figure 1a), although mean tree diameter (DBH) was slightly narrower in TZ site (Figure 1b). No significant differences were observed between mean BAI comparing the periods 1900–2018 (Figure 1c; Table S1) and 1961–2018 (Figure 1f; Table S1), while the BAI trend was positive in TZ and significantly different to TA (almost steady) during the periods 1900–2018 (Figure 1d; Table S1). Recent BAI trends (1961–2018) were slightly negative and not different between sites (Figure 1g; Table S1); however, growth variability, estimated as the CV, was significantly higher in the TZ site all over the studied period (Figure 1e,h; Table S1).

**Figure 2:**
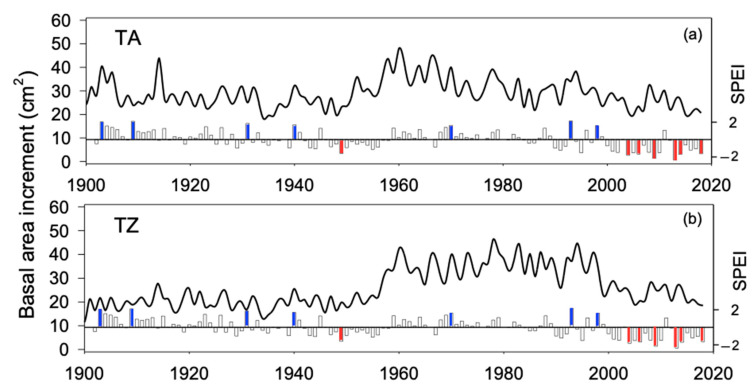
Growth pattern estimated as the basal area increments of tree rings obtained in *Abies marocana* for Talassemtane, TA (**a**) and Tazaot, TZ (**b**) over the period 1900–2020. Lines represent the mean. Bottom bars indicate the summer SPEI, where extreme moist (blue) and drought (red) events are highlighted.

**Figure 3:**
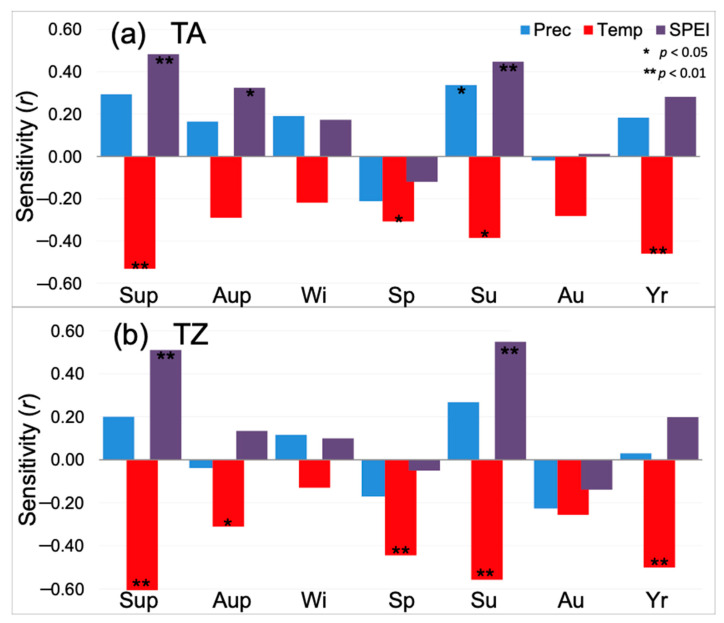
Climate sensitivity (correlation) of mean growth (mean basal area increment) for the period 1961–2018 of *Abies marocana* trees from Talassemtane, TA (**a**) and Tazaot, TZ (**b**) ranges. Sensitivity was tested at seasonal and annual scales for mean temperature (red), total precipitation (blue), and the SPEI drought index (purple). Seasonal scale was defined by mean (sum) of three-monthly scale temperature (precipitation), while seasonal-scale SPEI data were directly obtained from the database using the available three months estimates (noted as SPEI_3); similarly, annual estimates consider the hydrological year (prior September to current August), and the August estimate of SPEI_12 (Yr). Seasons are noted as winter (Wi), spring (Sp), summer (Su), and autumn (Au), including December–February, March–May, June–August, and September–November, respectively. Further, we consider seasonal correlations for summer and autumn of the previous year of tree-ring formation (Sup and Aup, respectively). * and ** indicate significant correlations after Bonferroni correction at a threshold of *p* < 0.01 and *p* < 0.05, respectively.

The growth pattern (Figure 2) was similar in both study sites (r = 0.78; *p* < 0.01) for the period 1961–2018. As a result, the climate sensitivity of the mean growth (mean basal area increment) was also alike in TA and TZ, supporting a significant sensitivity to summer climate conditions (Figure 3). Mean BAI showed positive correlations with water availability (SPEI) and negative correlations with the temperature of prior and current summer (Sup and Su, respectively; Figure 3). Spring (Sp) and year (Yr) were also significant (negative correlations) in both study sites, while SPEI during the prior autumn (Aup) and summer precipitation were significant (positive correlations) only in TA; temperature during the prior autumn was significant (negative correlation) only in TZ.

### 2.2. Genetic Structure of Populations

**Figure 4:**
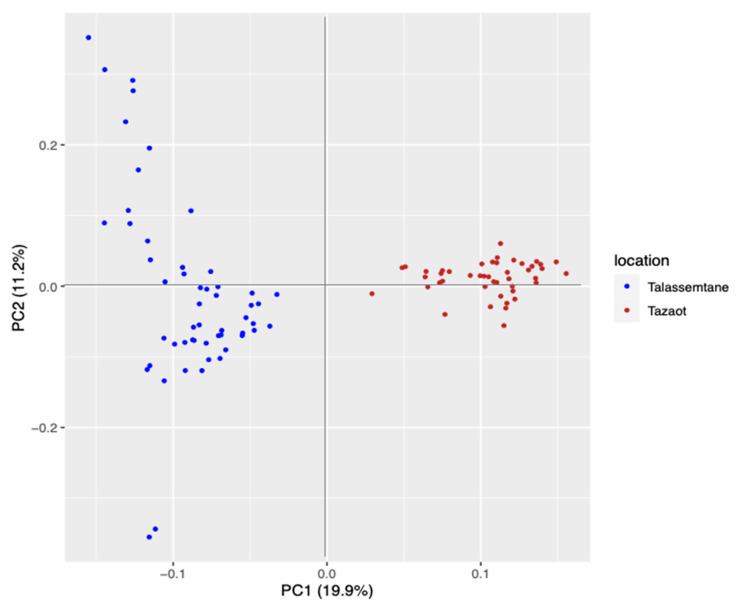
PCA results obtained comparing the two *Abies marocana* study sites. Talassemtane site is colored in blue, and Tazaot in brown. The axis of the two principal components (PC) and the percentage of variance explained by each axis are shown.

The vcf file obtained in the assembly was filtered, and a total of 98 individuals and 6131 SNPs were maintained. PCA showed two groups: one formed by Talassemtane and the other one by Tazaot (Figure 4). The percentage of explanation of this PCA was 19.9% in the first axis. It is noteworthy that the individuals from Talassemtane displayed high dispersion, while those of Tazaot were more similar to each other. The cross-entropy analysis indicated that K = 2 is the best explanation for the genetic structure of these populations, and the admixture study corroborated this result.

However, the AMOVA (Table S2) indicated low differences among populations, with 2% of the total, while the highest differences were observed within individuals, 57%. These results are based on the F_ST_ value (0.018), which showed that there are no differences between populations with a high significance (*p* = 0.0001). The number of private alleles was higher in Tazaot than in Talassemtane. Shannon index values were low but slightly higher in Tazaot. For Talassemtane and Tazaot, the grand mean values of the number of effective alleles are 1.524 and 1.528, respectively. Nei’s genetic distance (0.021) indicates that both populations are very close. Finally, the Nm value (13.393) shows the presence of a high gene flow among populations, which reduces population differentiation. Therefore, both populations are in complete panmixia.

### 2.3. Selection Signatures

The dataset showed 20 *loci* under selection. The 10 most significative SNPs were used to find homologies. Eight matches were obtained for nucleotide sequences, and five of them were similar to some proteins (Table S3). Among them, a peroxidase 5-like protein is the most interesting one, which is involved in environmental stress and oxidative stress responses. To assess the differences between old trees and saplings in the Moroccan dataset, another BayeScan study was carried out. In this case, 72 *loci* under selection were obtained, of which 20 are the same as those obtained in the BayeScan analysis with populations of Morocco without age separation. The 16 sequences most significative, of which 9 were used to find matches with Moroccan populations previously, were queried against the nucleotide database, and 14 matches were found. Finally, seven of them could be defined at the protein level (Table 3).

### 2.4. Genotype–Phenotype Associations (GPA)

**Figure 5:**
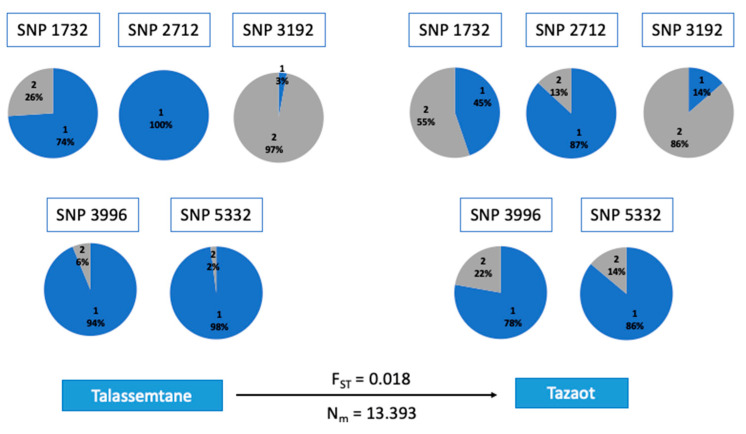
SNPs that showed associations with dendrophenotype variables, specifically with DBH (SNP 1732) and CV BAI 1900–2018 (SNP 2712, SNP 3192, SNP 3996, SNP 5332). The percentage of each allele is shown: blue for the reference allele and grey for the alternative. The fixation index (F_ST_) and migration rate (Nm) between the two sites are indicated above and below the arrow. The 4-digit number used as ID for the SNPs indicates the position in our genetic matrix.

The GPA study showed 11 associations with dendrophenotype variables related to tree-level growth patterns (see Table 4 for details): CV 1900–2018, CV 1961–2018, DBH, and BAI trend 1961–2018. CV 1900–2018 was associated with eight *loci*, DBH and BAI trend 1961–2018 with two *loci*, and CV 1961–2018 with one *locus*. Conversely, we did not find significant associations with tree-level climate sensitivity. As regards potential homologies, 16 matches were found against the nucleotide database, and 5 of them obtained hits for protein homology. Some of the proteins identified are related to ethylene biosynthesis (SNP 3192) or RNA binding (Table S4). Several allele frequencies of these SNPs associated showed differences between sites (Figure 5).

## 3. Discussion

The lack of genetic differences between sites is supported by intra-specific synchrony in growth patterns, although growth variability (CV) was significantly related to SNPs subjected to selection. Synchrony in growth response to climate (that is, climate sensitivity) supports similar climate-induced selective pressures in old trees over the study area [31]. Our results also suggest an increasing growth synchrony among trees during the last century (here estimated by the among-trees BAI inter-correlation), according to increasing warming, as has been recently reported over wide spatial and temporal scales (see [34] and references therein). Intra-specific synchrony in annual tree growth, both between and within sites, reflects a species-specific dependence on environmental variability in *A. marocana*. Here, TA and TZ mean growth shows a correlation of 0.78 for the period 1961–2018, while mean correlations among neighboring trees were 0.60 and 0.53 in TA and TZ, respectively, for the period 1961–2018. This covariance of the year-to-year variability in the growth of individual neighboring trees should be expected to intensify due to climate warming and drying conditions, supporting that within-population growth synchrony may be used as an integrative ecological measure of environmental stress, as well as to forecast effects of global climate change on tree growth [34,35].

It is widely acknowledged that tree-ring analysis provides past forest growth conditions at the annual resolution, as well as several important insights on tree sensitivity and adaptive capacity [9,10,11]. However, studies with natural populations which combine genomic and dendrophenotype data at the individual level are still scarce, i.e., [29,30,31,32,33,36]. Nevertheless, some results indicate that susceptibility to drought could be determined at the genomic level. For instance, the analysis of dendroecological and genetic data (SNPs in candidate genes) from *Abies alba* Mill. revealed that dendrophenotypes can be a powerful resource for genetic association studies [29]. Hence, fifteen genes were associated with the dendrophenotypes, including genes linked to photosynthesis and drought stress [29]. Additionally, by combining SNPs and dendrochronology, a study performed on *Nothofagus dombeyi* (Mirb.) Blume forests from northern Patagonia provided genetically based individual tree vulnerability to drought [30]. Indeed, this study found a set of 33 adaptive SNPs by comparing healthy and drought-induced declining trees, 8 of which were related to water stress. Further, association analysis between genomic variants and dendrophenotypic traits yielded six SNPs that were associated with growth patterns [30]. Another study, combining dendrochronology and population genetic analyses performed on *Nothofagus macrocarpa* [(DC.) Vásquez et Rodr.], found no relationship between growth patterns and genetic diversity [33]. In this case, tree genetic variability was estimated by nuclear microsatellite markers, which might reflect the limitations of methods such as simple sequence repeats (SSR) markers to account for genotype–phenotype associations [27,28]. Nonetheless, the use of individual tree-level dendrophenotypes in genetic association studies would greatly benefit our understanding of the response of trees to environmental stressors over time [31].

As regards the relict forests of *A. marocana,* dendrochronology has revealed past forest growth conditions, providing significant clues on forest dynamics [16]. Conversely, genomic studies on Moroccan fir were mainly focused on diversification, genetic diversity, and inter-specific relationships, i.e., [20,37,38]. Notwithstanding, the ability to identify genes or genomic regions related to adaptation to climate requires the evaluation of traits, such as climate–growth sensitivity, that precisely reflect the extent to that climate exerts a selective pressure [31,36]. Hence, the development of new techniques based on NGS technology, such as ddRAD-seq, allows the development of new molecular markers and describes the genetics of this species. To our knowledge, this work is the first to combine ddRAD-seq data and dendrophenotypes on this tree species. It is highlighted that our study was carried out using a high number of new molecular markers developed in the same study. These findings contrast with previous studies, which used molecular markers described formerly in other related species [17,18,19,20,38].

The taxonomy of the two studied sites has been under debate, sometimes stating the existence of two species, namely *A. marocana* and *A. tazaotana*, in TA and TZ, respectively [12]. Our results support that the Moroccan fir populations act as one population, meaning that these nuclei are in panmixia. Notwithstanding, the PCA and cross-entropy analyses have distinguished two groups, suggesting some genetic differences among them. Previous studies using SSRs concluded that there is no evidence to distinguish *Abies tazaotana* at the species level [19,20]. Recently, a genomic study based on RAD-seq technology, including several species of the *Abies* genus, also concluded that the Moroccan fir represents a single species [37].

TA showed a high scattering in the PCA, which could be due to the spatial distance among sampled trees (about 4 km), where the elevation ranges from around 1600 m to nearly 1800 m. Conversely, the study site of TZ covers less surface, and it was always close to 1700 m in elevation, while the distance among sampled trees was not much more than 1 km. Nevertheless, both sites showed similar climate sensitivity, supporting comparable environmental conditions and pointing out the relevance of the summer conditions as the main limiting season for drought-sensitive trees [9]. Hence, tree BAI showed positive correlations with water availability and negative correlations with the temperature of the prior and current summer, which is precisely the season where warming trends are expected to become worsen within the Mediterranean basin [7]. This agreement between observed climate sensitivity and expected climate trends enhances the threatened condition of relict *A. marocana* forests, as has already been stated for the whole circum-Mediterranean firs and *A. alba* across its southern distribution limits in Spain, Italy, and Romania [10,11].

Summer drought is the main factor impairing tree radial growth, reducing productivity and triggering tree defoliation and mortality events in the Mediterranean forest ecosystems [4,8,15]. However, tree-level responses to drought vary according to specific physiological characteristics and local adaptations [1,2,3,13]. Indeed, several SNPs associated with growth variables showed contrasting allele frequencies in TA and TZ, respectively (Figure 5). These differences suggest that a given allele may be more advantageous for one population than the other, which might be related to local conditions. Nonetheless, both *A. marocana* study sites were sensitive to summer drought, as they reduced their radial growth according to the summer water shortage (Figure 3).

Although it is expected that warming and extreme climate events such as droughts will increase as a consequence of forecasted climate change [7], *A. marocana* harbors a significant genetic diversity [17,18,19,20,38]. According to BayeScan results, the large number of *loci* under selection is noteworthy. Regarding the results obtained by comparing saplings and old trees, it is remarkable that this dataset gave a higher number of loci under selection than the global one. So, there are significant differences between old and juvenile trees. This contrasting selection signature may reflect the fact that the studied sapling trees could have been subjected to a selective pressure, as they have been established under a warmer and dryer climate scenario (Table 1), compared to old trees, mainly established around the onset of the 19th century.

We found two SNPs under selection present in both study sites and both age groups that depict homology with a peroxidase 5-like protein and LRK1 protein. The peroxidase function is related to several responses to stress, such as drought, oxidative damage, or pathogen defense. Several studies reported that the activity of peroxidases is affected by drought stress, increasing its activity in the conifers’ needles to reduce the damage of reactive oxygen species (ROS) [39,40]. As peroxidases are often upregulated under drought conditions, the obtained SNP could be relevant for the survival of *A. marocana* in the upcoming drier climate scenario. On the other hand, the LRK family is involved in the development, pathogen defense, and abiotic stress responses [41]. Although we evaluated several traits that precisely reflect drought-induced selective constraints, many of these *loci* under selection did not match with the protein database checked by us. This shortcoming illustrates the need to dedicate more research efforts to take access, for instance, to the sequenced genome of more conifer tree species and to identify genes or genomic regions related to adaptation to climate [23,31].

Contrary to our expectations, we did not obtain associations between the dendrophenotypes defined by climatic sensitivity and SNPs, according to the GPA. It should be expected to find associations among individuals’ sensitivity to climate variables and the genome-wide single nucleotide polymorphisms. Indeed, several pieces of evidence reported here and by previous studies illustrate the extent to which temperature and precipitation drive tree growth and stand dynamics in *A. marocana* and relative species [15,16]. Then, the absence of associations with tree-level climate sensitivity might be due to the significant growth synchrony and shared climate sensitivity found overall among the sampled trees (Figure 2 and Figure 3). Furthermore, it is important to note that the spatial resolution and time series length of available local climate data do not allow a reliable characterization of the differences in micro-climate between TA and TZ study sites. As a consequence, the lack of relationships between phenotypes related to climatic sensitivity and SNPs might be related not only to the scale of the study or the inherent similarity of both study sites, but also to the quality of the meteorological data. Thus, the investigation of highly complex mountain landscapes appears limited by the geographic resolution of the available meteorological data. Notwithstanding, field data obtained by the data logger and local rainfall gauges suggest similar average temperatures, but slightly lower precipitation in TZ compared to TA (J.C. Linares, unpublished data; M. Lamrani Alaoui, personal communication).

Although variables related to climate sensitivity did not show associations in the GPA, tree size, growth trends, and growth variability did. Stem diameter (DBH) increases steadily over a tree’s lifespan, whereas disentangling the effects of stem size and age on growth is challenging, as both increase together [42,43]; nonetheless, some studies suggest that growth is most strongly influenced by tree size specifically, supporting that large trees are more sensitive to drought [2]. Here, we obtained two associations with DBH but none with age supporting a size effect. Previous studies have also shown differences in growth responses to drought mainly explained by tree size [33]. Moreover, the variability (here estimated by the CV) in tree growth provides an additional piece of evidence about tree growth response to stress related to the SNPs analyzed. Identifying how growth variability influences drought sensitivity is not straightforward [44]. When growth variability is driven by inter-annual climate fluctuations, we can expect a larger vulnerability to drought with the increase in growth variability, as can be observed in gymnosperms from dry regions whose growth is severely constrained by drought [4]. This is also supported by the negative relationship found between the BAI trend and its CV during the period 1961–2018 (r = −0.73, *p* < 0.001), as well as previous studies reporting a negative relationship between mean BAI and growth variability [44]. The variability in growth may also be related to land-use legacies such as logging and pollarding, which poses an intriguing question regarding the observed relationship between tree genotypes, that is, the differential SNPs obtained according to growth variability and forest management. In summary, the complex picture found in this study suggests that further studies considering how growth variability is related to vulnerability to drought and land-use legacies, at the individual level, are clearly required [4,5,6,14].

All in all, the two nuclei studied here depict a single population from a genetic point of view, although some differences can be stated (Figure 4). Regarding the selection signatures, our results support enhanced selective pressures within the saplings group. In addition, our results support a negative effect of rising temperature and drought events on the secondary growth of old *A. marocana* trees, as has also been reported worldwide from several drought-sensitive forests [4,5,6]. Tree growth decline and dieback are predicted to increase even further, while understanding the genetic basis of tree adaptive capacity to changing environments is essential to the conservation of relict and endangered species [14,23]. Here, the combined analysis of genome-wide single nucleotide polymorphisms and dendrophenotypes provided genetically based evidence of tree-level sensitivity to climate, as well as preliminary evidence of climate change-induced selection in sapling cohorts. Nonetheless, the molecular mechanisms of adaptation remain largely unknown for long-lived tree species [23]. Finally, we would point out future research lines, such as the newly developed methods to quantify the risk of non-adaptedness, which can predict the genetic offset or genomic vulnerability of species via allele frequency change under multiple scenarios of climate change [36].

## 4. Materials and Methods

### 4.1. Study Sites

This study was carried out in the mountain range of Talassemtane and in Jebel Tazaot (both included in Talassemtane National Park). The range occupied by this fir in Talassemtane (TA) is around 3760 ha, while the range of Tazaot (TZ) extends over approximately 300 ha, where fir grows mostly on northern slope [45,46,47].

*A. marocana* is a monoecious or sub-dioecious conifer that grows between 1500 and 2000 m a.s.l. [12] in mountains near the coast, where annual rainfall fairly exceeds 1000 mm, and is a drought-sensitive species [13,14,15,16]. In the lower elevation limits of dense *A. marocana* forests, the vegetation is Mediterranean, dominated by *Quercus rotundifolia* Lam. (holm oak), *Quercus faginea* Lam. (gall oak), *Pinus pinaster* Ait. (Maritime pine), and *Pinus halepensis* Mill. (Aleppo pine) forests. Upslope, *A. marocana* is the dominant tree to roughly 1700 m, where it grows with other relict trees, such as *Acer granatense* Boiss. (Spanish Maple), *Taxus baccata* L. (yew), *Cedrus atlantica* Manetti (Atlas cedar), and *Pinus nigra salzmannii* (Dunal) Franco (Laricio pine).

The Moroccan fir is endemic and relict, limited to the Rif mountains of North Morocco; this fir is included in the IUCN Red List of Threatened Species as endangered species [48], and one of the most serious hazards for its survival is climate change. Soils are usually shallow, rocky, and developed on limestone.

The mean temperature range in the study area goes from 12 to 14 °C, with a maximum of 33 °C, and a minimum of 0 °C, reaching −3 °C at high elevation. The mean annual precipitation is above 500 mm and can exceed 2000 mm at high mountain peaks [49]. As a whole, the rainfall patterns are distinctly Mediterranean, with approximately 90% of all precipitation falling between October and April, followed by a long summer drought.

### 4.2. Climate Data

The spatial resolution and time series length of available local climate data do not allow us to characterize the differences in micro-climate between Talassemtane and Tazaot study sites. Notwithstanding, field data obtained by data logger (HOBO Onset) and local rainfall gauges provided that mean annual temperature is about 10.1 °C and 10.5 °C, while total annual precipitation is about 1821 mm and 1644 mm, in Talassemtane and Tazaot, respectively (J.C. Linares, unpublished data; M. Lamrani Alaoui, personal communication). As a consequence, we performed climate–growth relationships using the same climate dataset in both study sites, using the Climate Explorer webpage (https://climexp.knmi.nl/). Monthly mean temperature (T, units in °C; 0.25° resolution) and total monthly precipitation (*p*, units in mm; 0.25° resolution) were downloaded from the EOBS database v23.1e for the period 1921–2021 [50], available at the Climate Explorer webpage (https://climexp.knmi.nl/). Alike, to quantify drought severity, we used the Standardized Precipitation Evapotranspiration Index (SPEI), considering extreme drought events’ annual SPEI values below −1.5 [51]. The SPEI is a multiscalar drought index based on climatic data. It can be used for determining the onset, duration, and magnitude of drought conditions with respect to normal conditions in a wide variety of ecosystems. The SPEI is calculated using the monthly difference between precipitation and evapotranspiration. This difference provides a measure of the water surplus or deficit for the analyzed month. Then, precipitation minus evapotranspiration values are aggregated at different time scales, providing seasonal, annual, or multiannual estimates. Finally, the estimate is standardized following a log-logistic distribution [51].

Climate variables were estimated based on monthly, seasonal, and annual estimates of mean temperature, total precipitation, and SPEI. In order to consider seasonal variations of BAI sensitivity to climate, the climatic variables were aggregated at the three-monthly scale, thus defining four seasons, winter, spring, summer, and fall, as December–February, March–May, June–August, and September–November, respectively. Further, we consider these seasonal correlations for summer and autumn of the previous year of tree-ring formation to test the effect of prior climate conditions on current growth. Finally, the effect of yearly average was also tested. Note that winter was not discarded from the analysis as growth and photosynthesis can be substantially influenced by climatic conditions during this period and are often ongoing in hotter years. Temporal trend of seasonal/annual mean temperature and total precipitation were estimated using the Mann–Kendall test (hereafter MK) [52,53]. MKs are non-parametric tests for the detection of trend in a time series. These tests are widely used in environmental science, because they are simple, robust and can cope with missing values and values below a detection limit. For each time series element *xi* (1, 2,…, n), the number *ni* of *xj* elements lower than *xi* (*xj* < *xi*) previous (*j* < *i*) are counted. Then, the MK statistic for the time series {Zk, k = 1,2,…, n} is defined as

(1) $$T=\sum_{j<i}\mathrm{sgn}(Zi-Zj)$$

where

$$\mathrm{sgn}(x)\left\{ \begin{array}{l} 1,\_if\_x>0\\ 0,\_if\_x=0\\ -1,\_if\_x<0 \end{array}\right.$$

If no ties between the observations are present and no trend is present in the time series, the test statistic is asymptotically normally distributed with mean *E(T)* = 0, independent of the distribution function of the data. The null hypothesis assumes that the trend is zero, and it is rejected if *E(T)* is higher in absolute value than 1.96 for a two tails test at 95% significance, or 2.58 at 99% of significance. The *E(T)* sign indicates if the trend is positive or negative [52,53]. We computed climate trends for the period 1921–2021. Further, we computed temporal climate trends and the climate–growth sensitivity from 1961 onward, based on the reference period adopted by the IPCC [54], which provides a standard reference for many climate change impact studies.

### 4.3. Field Sampling and Dendrochronological Methods

**Figure 6:**
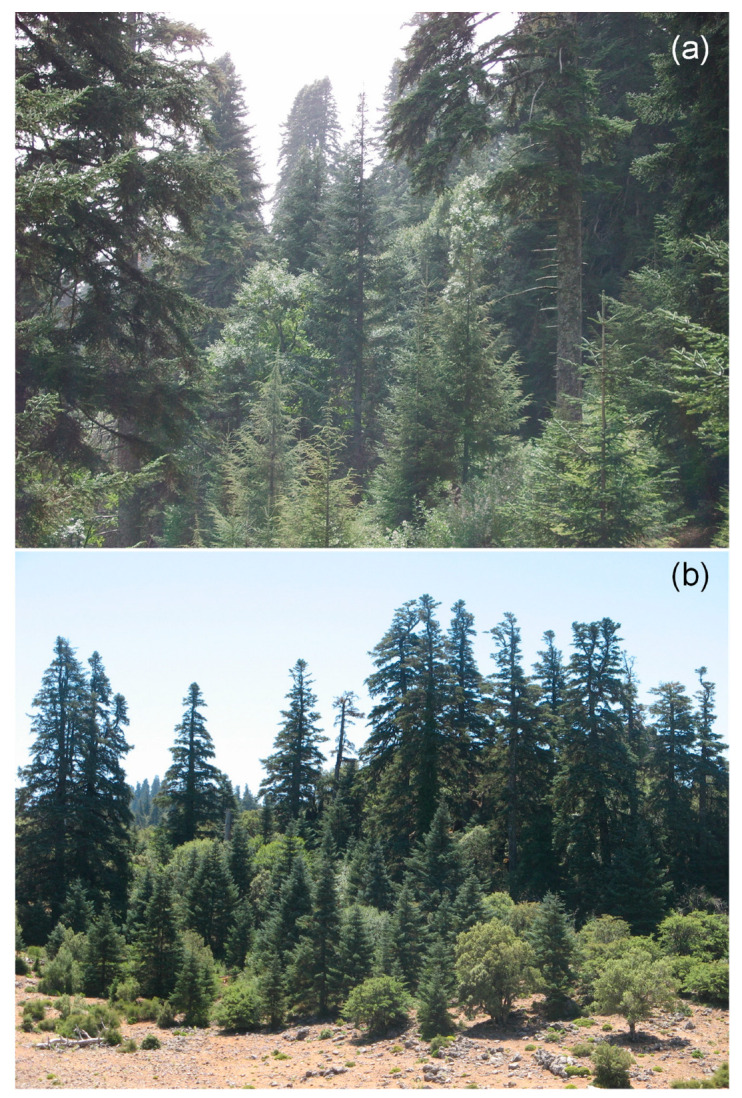
High tree species diversity in *Abies marocana* forest of Talassemtane (**a**) and Tazaot (**b**) ranges, north Morocco. Photo: Juan Carlos Linares.

Field sampling was carried out at Talassemtane (TA, 35.14 N, −5.14 W, 1653 m a.s.l.) and Tazaot (TZ, 35.26 N, −5.10 W, 1722 m a.s.l.) ranges, which are home to natural unevenly aged *Abies marocana* stands that have not been intensively managed since the middle 20th century [12] (Figure 6).

Field sampling was focused on old trees (about 200 years old) and close saplings (about 15 years old) to investigate selection signatures between cohorts. To determine tree age, we took advantage of the preformed-growth pattern shown by *Abies marocana* (regarding the saplings) and previous dendrochronological studies (regarding the old trees) [16]. In this species, each continuous and unique annual growth portion in the main stem and lateral branches of trees is separated (and is thus easily identifiable) by permanent scars. Such scars are left as marks after the fall of apical bud scales, once primary growth (stem and branches elongation) is resumed at the beginning of each year’s growing season. As regards the old trees, we performed previous dendrochronological studies allowing us to identify old trees in the field. Further, we validated the selected old trees in situ by sampling wood cores and performing a preliminary inspection of the tree rings. In late autumn 2018, we sampled 25 mature, dominant, and healthy old trees (wood cores and needles), 25 close saplings (needles) in TA site, and 20 old trees and 20 saplings in TZ site. Old trees were cored to the pith at breast height perpendicularly to the slope. Each tree was measured for trunk circumference and bark thickness. Bored cores were 5 mm in diameter; at least three cores per tree were extracted, and pith was reached in at least one of each set. Dendroecological analysis was conducted for all cores for age determination and radial growth measurement.

The cores were sanded until the tree rings were clearly visible under a binocular microscope. All samples were visually cross-dated. Tree ring widths were measured to 0.01 mm using a LINTAB measuring device (Rinntech, Germany), and the cross-dating quality was checked using COFECHA [55]. Growth patterns were estimated as the basal area increments (BAI) of tree rings for a more accurate reflection of annual radial growth around the circumference of the tree. Tree age was estimated at coring height (roughly 1.3 m from the ground), as well as the tree diameter (DBH). The coefficient of variation (CV) was estimated as the ratio of the BAI standard deviation divided by the mean BAI and expressed in %. BAI trends were estimated as the slope of BAI against the time. Within-tree BAI autocorrelation was estimated as the first-order correlation of the BAI_(*i*)_ and the BAI_(*i*+1)_. Among trees, BAI inter-correlation was estimated as the correlation of the individual BAI time series with the mean BAI time series. Differences between sites for tree age, DBH, mean BAI, CV, BAI trend, within-tree BAI autocorrelation, and among-trees BAI intercorrelation were compared by ANOVA using a significance level of *p* < 0.05. Climate sensitivity was estimated by the Pearson correlation coefficient (*r*), with significance corrected using Bonferroni adjustment [56]. All the analyses were calculated using R software [57].

### 4.4. DNA Extraction and ddRAD-seq

Fresh needle samples were collected in the same old trees and close saplings from the above-mentioned dendrochronological sampling. Then, 100 mg of each sample was lyophilized, and total DNA extraction was carried out by using DNeasy Plant Mini Kit (Qiagen^®^, Berlin, Germany) following the manufacturer’s instructions with some modifications. DNA concentration was measured on a NanoDrop™ spectrophotometer, and the integrity of the samples extracted was determined by electrophoresis in 1% agarose gel.

Subsequently, ddRAD-seq [21] libraries were constructed using *Ape*KI/*Pst*I double digestion and sequenced by LGC Genomics (Berlin, Germany). ddRAD-seq is an NGS technique that can be used with large genomes and in absence of a reference genome. This technique allows us to obtain high number of molecular markers, especially single nucleotide polymorphisms (SNP), and to carry out several kinds of studies, such as genome–environment and genotype–phenotype association studies [21]. One of the main advantages of using restriction enzymes is the reduction in genome complexity due to fragmentation. *Ape*KI (5′G/CWGC3′), a methylation-sensitive enzyme, is blocked by overlapping CpG methylation, which implies that it avoids cutting the methylated regions present in the genomes. As the presence of repetitive sequences in conifers genomes is very common, and generally heavily methylated, the use of this enzyme reduces the presence of this kind of sequences in ddRAD-seq libraries. On the other hand, *Pst*I (5′CTGCA/G’3) is not sensitive to methylation, but it is a rare cutter enzyme. Therefore, the combination of both enzymes increases representation of coding regions [58] and, subsequently, the possibility of sequencing the same fragments in different samples.

The reads obtained were paired-ended with a depth of 1 M. FastQC v0.11.9 [59] was used to determine the quality of the raw reads. Subsequently, adapter sequences were trimmed, and those sequences which were low-quality were removed using fastp v0.12.4 [60]. A de novo assembly was carried out, and the SNP callings were performed using ipyrad v.0.9.65 [61]. Several filter steps were carried out until the obtention of the genetic matrix. All filtrations were performed using VCFtools v0.1.16 [62] with the aim to retain those SNPs which were biallelic, with a minimum allele frequency (MAF) of 5% and maximum missingness of 50%; to avoid the linkage disequilibrium, we retain only 1 SNP per *locus*. Individuals with less than 50% of the filtered SNPs were removed. Once the SNP matrix was created, several analyses were carried out using it.

### 4.5. Genetic Structure of Populations

Two approaches to studying the genetic structure of the populations were performed: principal component analysis (PCA) and sparse non-negative matrix factorization analysis (sNMF). PCA was carried out using the plink2 2.00a2.3 software [63] with the --pca option, and then, a graphic representation was obtained using the R v4.1.2 [56] package called ggplot2 v3.3.5 [64]. For the second approach, a cross-entropy study was carried out using the snmf function of the LEA package v3.4.0 [65] in R software v4.1.2 [56] with the aim to obtain the most probable number of ancestry populations (named K) that best explains the structure of the populations. For this analysis, a total of 10 repetitions for each K ranging from 1 to 4 was performed. In addition, the admixture coefficients were obtained and then employed to obtain a graphic representation of the admixture results using pong [66]. Several statistical analyses were performed using the GenAlEx v6.5 software [67,68]. Parameters, such as fixation indexes (F_ST_), to determine population differentiation, and migration rate (Nm), to describe presence or not of gene flow among populations, were estimated. The Nei’s genetic distance between populations was also estimated. Shannon index was inferred to describe genetic diversity. In addition, F_ST_ coefficient was used to perform a molecular variance analysis (AMOVA) with the aim of determining the proportion of genetic variation attributable to differences among and within populations. A total of 9999 permutations were used to carry out the AMOVA.

### 4.6. Selection Signatures

Selection signatures in the genetic matrix were detected using BayeScan 2.1 software [69]. Default parameters were applied. This software calculated the F_ST_ coefficients for each locus and compared them among and within the populations of study with the aim of determining differences. This version of BayeScan directly calculates q-values using the false discovery rate (FDR) correction. In this study, the threshold was established in 5%, so all SNPs with a q-value < 0.05 were significant to be under selection. To know more about the genetic variants under selection, the sequences which contain the SNPs were queried against the nucleotide database, and in the case of the matrixes obtained from reference assembly, against transcriptome shotgun assembly (TSA) database using, in both cases, BLASTn (NCBI) [70]. When a match was obtained, the sequence was used to perform a BLASTx (NCBI) [71] against the non-redundant protein database, with the aim of gaining knowledge about the protein which could be derived from the SNP scaffold.

### 4.7. Genotype–Phenotype Associations (GPA)

To determine the existence of associations between dendrophenotypes and the SNPs obtained, a genome–phenotype associations study was carried out. Since the phenotype data were only available for old trees, a reduced genetic matrix consisting of 44 individuals and 6029 SNPs was used for GPA. Dendro-phenotypic traits included both tree-level growth patterns and climate sensitivity (Table 3)**.** The rrBLUP v4.6.1 R package was used to perform GPA [72,73]. To obtain q-values, FDR correction was applied to *p*-values estimated in the GPA, and only those associations with a significance of 5% were used to find homologies with nucleotide sequences and proteins in NCBI databases.

## Figures and Tables

**Table 1:** Temporal trends observed in seasonal and annual total precipitation and mean temperature according to Mann–Kendall statistic for the period of 1921–2021 and 1961–2021. Seasons are noted as winter (Wi), spring (Sp), summer (Su), and autumn (Au), including December–February, March–May, June–August, and September–November, respectively. Annual estimates consider the hydrological year (prior September to current August). Mean change in each variable over the considered time span was computed, only if the slope was statistically significant, by multiplying the slope estimate (mm or °C year ^−1^) per the number of years.

Time Span	1921–2021					1961–2021			
Variable	Season	Mean (mm, °C)	Mean Change (Slope*Time Span; mm, °C)	MK-Stat	*p*-Value	Mean (mm, °C)	Mean Change (Slope*Time Span; mm, °C)	MK-Stat	*p*-Value
Total precipitation	Wi	468	165	2.06	0.0394	521	ns	−1.23	0.2179
	Sp	291	ns	0.76	0.4490	299	ns	0.10	0.9207
	Su	23	−30	−2.67	0.0076	20	−26	−2.94	0.0032
	Au	322	ns	1.59	0.1118	337	ns	0.58	0.5586
	Yr	1105	219	2.05	0.0406	1178	ns	−0.83	0.4044
Mean temperature	Wi	7.7	2.4	7.28	<0.0001	8.1	1.8	4.47	<0.0001
	Sp	11.2	0.8	3.09	0.0020	11.1	2.0	5.86	<0.0001
	Su	18.5	ns	−1.24	0.2145	18.2	1.7	4.34	<0.0001
	Au	14.4	ns	1.48	0.1392	14.4	1.5	3.81	0.0001
	Yr	12.9	0.9	3.50	0.0005	13.0	1.7	5.71	<0.0001

**Table 2:** Location and growth pattern estimated as the basal area increments (BAI) of tree rings obtained in *Abies marocana* for Talassemtane and Tazaot. Tree age was estimated at coring height, according to tree-ring dating. DBH indicates the tree diameter at 1.3 m from the ground. CV indicates the coefficient of variation, estimated as (100*) the ratio of the BAI standard deviation divided by the mean. BAI trend was estimated as the slope of BAI against the time. Within-tree BAI autocorrelation was estimated as the first-order correlation of the BAI_(*i*)_ and the BAI_(*i*+1)_. Among trees, BAI inter-correlation was estimated as the correlation of the individual BAI time series with the mean BAI time series. Values are mean ± standard error. * indicates significant differences (ANOVA, *p* < 0.05).

	Talassemtane	Tazaot
Latitude (°N)	35.14 ± 0.006	35.26 ± 0.001
Longitude (°W)	−5.14 ± 0.001	−5.10 ± 0.001
Elevation (m a.s.l.)	1652.80 ± 23.890	1722.33 ± 10.423
Tree age (years)	221.72 ± 12.274	214.95 ± 11.459
DBH (cm)	84.44 ± 3.066	73.18 ± 2.821 *
BAI mean 1900–2018 (cm^2^)	28.97 ± 2.109	26.07 ± 2.177
CV 1900–2018 (%)	39.60 ± 2.379	55.03 ± 5.568 *
BAI trend 1900–2018 (cm^2^year^−1^)	−0.01 ± 0.031	0.12 ± 0.042 *
Within-tree BAI autocorrelation 1900–2018	0.71 ± 0.028	0.76 ± 0.035
Among-trees BAI intercorrelation 1900–2018	0.50 ± 0.033	0.49 ± 0.074
BAI mean 1961–2018 (cm^2^)	29.75 ± 2.355	31.62 ± 3.124
CV 1961–2018	31.29 ± 2.232	39.92 ± 3.343 *
BAI trend 1961–2018 (cm^2^year^−1^)	−0.25 ± 0.069	−0.28 ± 0.102
Within-tree BAI autocorrelation 1961–2018	0.58 ± 0.038	0.68 ± 0.030
Among-trees BAI intercorrelation 1961–2018	0.60 ± 0.039	0.53 ± 0.065

**Table 3:** BayeScan results of de novo assembly obtained for *Abies marocana* separating between saplings and old trees in both study sites. For the identified SNPs, the sequence type, the name and functions of the proteins, and the E-value are shown (lower values indicate increasing probability for protein similarity). Protein functions were obtained from UniProt. The 4-digit number used as ID for the SNPs indicates the position in our genetic matrix.

SNP ID	Sequence Type	Protein Name	Protein Function	E-Value
1127	Transcribed RNA sequence *Abies pinsapo*	Formyltetrahydrofolate deformylase 1 (mitochondrial)	Biosynthesis of purines Metabolism of amino acids	2 × 10^−104^
1369	mRNA *Picea glauca*	Disease resistance protein	Pathogen response (viruses, bacteria, or fungi)	4 × 10^−58^
2458	mRNA *Picea glauca*	Peroxidase 5-like	Response to environmental stress, pathogen, and oxidative stress Auxin catabolism Suberization	5 × 10^−65^
2769	mRNA *Picea glauca*	Alpha-D-phospohexomutase superfamily	Catalyze a phosphoryl transfer on sugar substrates	0.0
3755	Transcribed RNA sequence of *Picea glauca*	WD-40 repeat family protein	Signal transduction Protein trafficking Transcriptional mechanisms	2 × 10^−5^
5262	mRNA *Pinus taeda*	Clavata 1-like protein	Cell differentiation Meristem structure regulation Peptidyl-serin autophosphorylation	8 × 10^−9^
5594	mRNA *Picea glauca*	LRK1	Protein phosphorylation	2 × 10^−151^

**Table 4:** Dendrophenotypic traits used in the genotype–phenotype associations (GPA) to relate tree-level growth patterns and climate sensitivity with the single nucleotide polymorphisms (SNPs) obtained.

Variable Group	Abbreviation	Description (Units)
Tree-level growth patterns	Age	Tree age at coring height (years)
	DBH	Tree diameter at 1.3 m from the ground (cm)
	BAI mean 1900–2018	Mean basal area increment for the indicated time span (cm^2^)
	BAI trend 1900–2018	Slope of the basal area increment over time (calendar year) for the indicated time span (cm^2^year^−1^)
	Within-tree BAI autocorrelation 1900–2018	Tree-level first-order autocorrelation of the basal area increment over time (calendar year) for the indicated time span (Pearson’s correlation coefficient)
	CV 1900–2018	Tree-level coefficient of variation of the basal area increment (quotient of the standard deviation divided by the mean, multiplied per 100) for the indicated time span (%)
	Among-trees BAI inter-correlation 1900–2018	Correlation between the tree-level basal area increment and population mean basal area increment for the indicated time span (Pearson’s correlation coefficient)
	BAI mean 1961–2018	As above, for the indicated time span
	BAI trend 1961–2018	
	Within-tree BAI autocorrelation 1961–2018	
	CV 1961–2018	
	Among-trees BAI inter-correlation 1961–2018	
Tree-level climate sensitivity (Pearson’s correlation coefficient of the tree-level basal area increment and the described climate variable for the time span 1961–2018)	p_sup	Total precipitation (P) of summer (June, July, August) prior to growing season (mm, for all P variables)
	p_aup	P of autumn (September, October, November) prior to growing season
	p_wi	P of winter (December, January, February) prior to growing season
	p_sp	P of growing season spring (March, April, May)
	p_su	P of growing season summer (June, July, August)
	p_au	P of growing season autumn (September, October, November)
	p_yr	Total annual precipitation (prior September to current August)
	t_sup	Mean temperature (T) of summer prior to growing season (°C, for all T variables); seasonal month’s intervals as above
	t_aup	T of autumn prior to growing season
	t_wi	T of winter prior to growing season (mm)
	t_sp	T of growing season spring
	t_su	T of growing season summer
	t_au	T of the growing season autumn
	t_yr	Mean annual T
	spei_sup	Standardized Precipitation Evapotranspiration Index (SPEI) of summer prior to growing season; seasonal month’s intervals as above
	spei_aup	SPEI of autumn prior to growing season
	spei_wi	SPEI of winter prior to growing season (mm)
	spei_sp	SPEI of growing season spring
	spei_su	SPEI of growing season summer
	spei_au	SPEI of growing season autumn
	spei_yr	Annual SPEI

## Data Availability

Not applicable.

## Supplementary Materials

The following supporting information can be downloaded at https://www.mdpi.com/article/10.3390/plants12040873/s1. Figure S1: Mean temperature and total precipitation (a), and SPEI (b) data of the study area for the period 1921–2020. Figure S2: Growth pattern estimated as the basal area increments of tree rings obtained in *Abies marocana* for Talassemtane (a) and Tazaot (b). Lines represent the mean, while points indicate raw measurements. Bottom bars indicate the sample size (number of trees with growth data for this year). Table S1: Results obtained by ANOVA comparing tree age, size, and growth patterns in Talassemtante and Tazaot populations. Table S2: Results obtained by AMOVA studies in Talassemtante and Tazaot populations. The rows indicate the partitions of genetic variability in two components: among and within populations. The columns show degrees of freedom (df), sum of squares (SS), mean squares (MS), estimate of variance (Est.var.), and the percentage of total variation (%). The percentage of variation is higher within individuals than among populations. Table S3: Results of the matches found in the alignments against protein database with those loci which were significant in BayeScan analysis comparing *Abies marocana* TA and TZ study sites for the identified SNPs; the sequence type, the name, and functions of the proteins, and the E-value are shown (lower values indicate increasing probability for the protein similarity). Protein functions were obtained from UniProt. The 4-digit number used as ID for the SNPs indicates the position in our genetic matrix. Table S4: Results obtained at protein level with those SNPs, which showed association with some variables in GPA studies. For the identified SNPs, the sequence type, the name and functions of the proteins, and the E-value are shown (lower values indicate increasing probability for protein similarity). Protein functions were obtained from UniProt. The 4-digit number used as ID for the SNPs indicates the position in our genetic matrix.
