# Supplementary material for: Tree-Level Growth Patterns and Genetic Associations Depict Drought Legacies in the Relict Forests of Abies marocana
Source: Plants (Basel). 2023 Feb 15;12(4):873. doi: 10.3390/plants12040873 (PMC9959318; doi:10.3390/plants12040873)
Supplement: Supplementary file 1 [file plants-12-00873-s001.zip › plants-2125966-SI.pdf]

plants

MDPI

Article

Tree-level Growth Patterns and Genetic Associations Depict Drought Legacies in the Relict Forests of *Abies marocana*

Belén Méndez-Cea [1], Isabel García-García [1], Raúl Sánchez-Salguero [2], Víctor Lechuga [3], Francisco Javier Gallego [1], and Juan C. Linares [2]*

[1] Dpto. Genética, Fisiología y Microbiología, Unidad de Genética, Facultad de CC Biológicas, Universidad Complutense de Madrid, 28040, Spain. belenmen@ucm.es (B. M-C); isabel06@ucm.es (I. G-G); fjgalleg@ucm.es (F.J.G)
[2] Dpto. Sistemas Físicos, Químicos y Naturales, Universidad Pablo de Olavide, 41013 Sevilla, Spain. rsanchez@upo.es (R.S-S), jclincal@upo.es (J.C.L.)
[3] Centro de Estudios Avanzados en Ciencias de la Tierra, Energía y Medio Ambiente (CEACTEMA). Universidad de Jaén. Jaén, Spain; vlechuga@ujaen.es (V.L.).
* Correspondence: jclincal@upo.es (J.C.L.)

SUPPLEMENTARY MATERIAL

Citation: Méndez-Cea et al. Tree-level Growth Patterns and Genetic Associations Depict Drought Legacies in the Relict Forest of *Abies marocana*. *Plants* **2023**, *12*, x. https://doi.org/10.3390/xxxxx

Academic Editor: Firstname Lastname

Received: date
Accepted: date
Published: date

Publisher's Note: MDPI stays neutral with regard to jurisdictional claims in published maps and institutional affiliations.

Copyright: © 2022 by the authors. Submitted for possible open access publication under the terms and conditions of the Creative Commons Attribution (CC BY) license (https://creativecommons.org/licenses/by/4.0/).

plants

MDPI

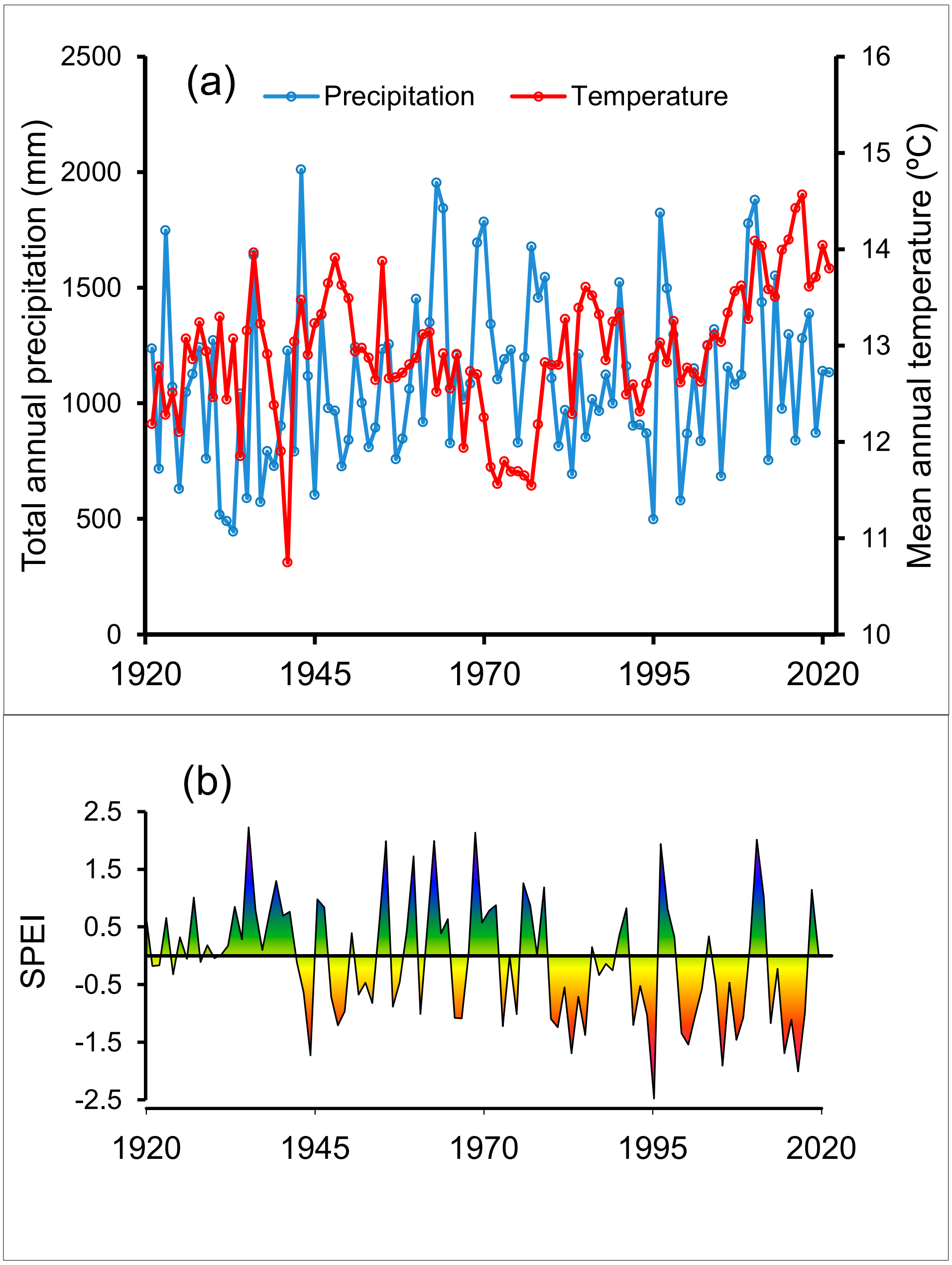

Figure S1. Mean temperature and total precipitation (a), and SPEI (b) data of the study area for the period 2021-2021.

plants

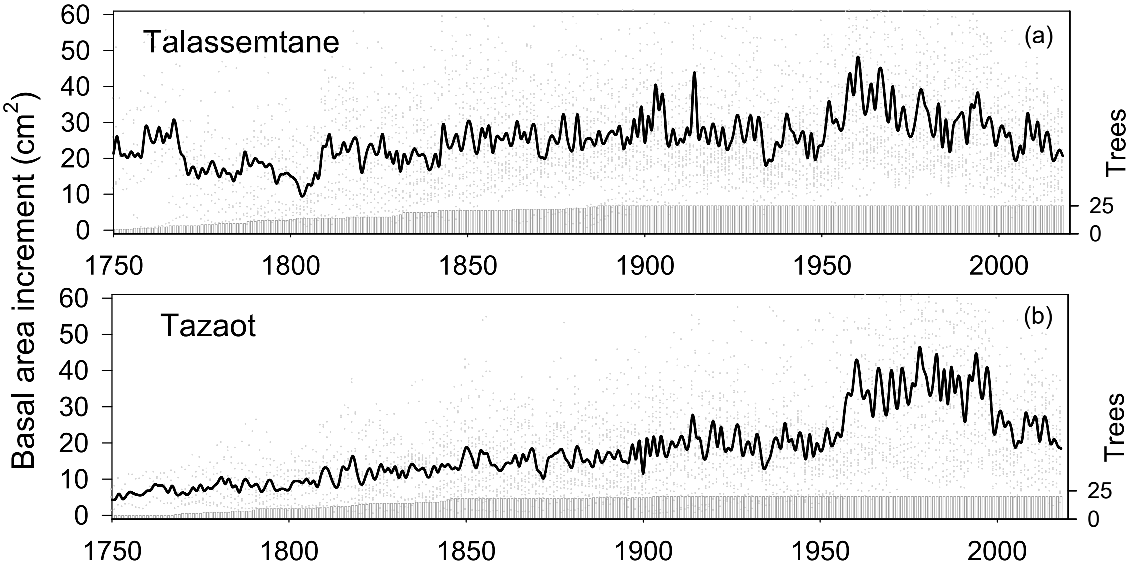

Figure S2. Growth pattern estimated as the basal area increments of tree-rings obtained in *Abies marocana* for Talassemtane (a) and Tazaot (b). Lines represent the mean while points indicate raw measurements. Bottom bars indicate the sample size (number of trees with growth data for this year).

Table S1. Results obtained by ANOVA comparing tree age, size and growth patterns in Talassemtante and Tazaot populations.

		Intercept	Population	Error	Total
Degree of Freedom		1	1	43	44
Age 1900-2018	SS	2118674.00	509.00	140284.00	140793.00
	MS	2118674.00	509.00	3262.00	
	F	649.418	0.156		
	p	0.000	0.695		
DBH 1900-2018	SS	276039.100	1410.600	8663.800	10074.400
	MS	276039.100	1410.600	201.500	
	F	1370.039	7.001		
	p	0.000	**0.011**		
BAI mean 1900-2018	SS	33663.850	93.010	4469.080	4562.090
	MS	33663.850	93.010	103.930	
	F	323.903	0.895		
	p	0.000	0.349		
BAI trend 1900-2018	SS	0.146	0.175	1.227	1.402
	MS	0.146	0.175	0.029	
	F	5.114	6.145		
	p	0.029	**0.017**		

BAI autocor 1900-2018	SS	23.960	0.035	0.958	0.993
	MS	23.960	0.035	0.022	
	F	1075.540	1.555		
	p	0.000	0.219		
CV 1900-2018	SS	99509.010	2643.860	15177.010	17820.880
	MS	99509.010	2643.860	352.950	
	F	281.932	7.491		
	p	0.000	**0.009**		
BAI intercor 1900-2018	SS	10.858	0.003	2.732	2.735
	MS	10.858	0.003	0.064	
	F	170.885	0.047		
	p	0.000	0.830		
BAI mean 1961-2018	SS	41839.350	38.800	7034.000	7072.800
	MS	41839.350	38.800	163.580	
	F	255.771	0.237		
	p	0.000	0.629		
BAI trend 1961-2018	SS	3.162	0.010	6.847	6.856
	MS	3.162	0.010	0.159	
	F	19.859	0.061		
	p	0.000	0.806		
BAI autocor 1961-2018	SS	17.663	0.093	1.197	1.290
	MS	17.663	0.093	0.028	
	F	634.488	3.332		
	p	0.000	0.075		
CV 1961-2018	SS	56353.260	827.310	7235.590	8062.890
	MS	56353.260	827.310	168.270	
	F	334.899	4.917		
	p	0.000	**0.032**		
BAI intercor 1961-2018	SS	14.295	0.061	2.538	2.599
	MS	14.295	0.061	0.059	
	F	242.177	1.027		
	p	0.000	0.317		

plants MDPI

Table S2: Results obtained in AMOVA studies with Talassemtante and Tazaot populations. The rows indicate the partitions genetic variability in two components: among and within populations. The columns show degrees of freedom (df), sum of squares (SS), mean squares (MS), estimate of variance (Est.var.) and the percentage of total variation (%). The percentage of variation is higher within individuals than among populations.

Summary AMOVA Table					
**Source**	**df**	**SS**	**MS**	**Est. Var.**	**%**
**Among Pops**	1	4515.948	4515.948	26.029	2%
**Among Indiv**	96	190280.027	1982.084	587.692	41%
**Within Indiv**	98	79056.500	806.699	806.699	57%
**Total**	195	273852.474		1420.421	100%

plants MDPI

Table S3: Results of the matches found in the alignments against protein database with those loci which were significant in BayeScan analysis comparing *Abies marocana* TA and TZ study sites. For the identified SNPs, it is shown the sequence type, the name and functions of the proteins, and the E-value (lower values indicate increasing probability for the protein similarity). Protein functions were obtained from UniProt. The 4-digit number used as ID for the SNPs indicates the position in our genetic matrix.

SNP ID	Sequence type	Protein name	Protein function	E-value
1127	Transcribed RNA sequence *Abies pinsapo*	Formyltetrahydrofolate deformylase 1 (mitochondrial)	Biosynthesis of purines<br>Metabolism of amino acids	2e-104
1369	mRNA *Picea glauca*	Disease resistance protein	Pathogen response (viruses, bacteria, or fungi)	4e-58
2458	mRNA *Picea glauca*	Peroxidase 5-like	Response to environmental stress, pathogen, and oxidative stress<br>Auxin catabolism<br>Suberization	5e-65
5262	mRNA *Pinus taeda*	Clavata 1-like protein	Cell differentation<br>Meristem structure regulation<br>Peptidyl-serin autophosphorylation	8e-09
5594	mRNA *Picea glauca*	LRK1	Protein phosphorylation	2e-151

plants MDPI

Table S4: Results obtained at protein level with those SNPs which showed association with some variables in GPA studies. For the identified SNPs, it is shown the sequence type, the name and functions of the proteins, and the E-value (lower values indicate increasing probability for the protein similarity). Protein functions were obtained from UniProt. The 4-digit number used as ID for the SNPs indicates the position in our genetic matrix.

SNP ID	Sequence type	Protein name	Protein function	E-value
196	mRNA *Picea glauca*	Nudix hydrolase 3-like	Regulatory and signaling roles in metabolism	3e-62
3192	mRNA *Picea glauca*	S-adenosylmethionine synthase	Ethylene biosynthesis	0.0
5531	mRNA *Picea glauca* (clone)	APO protein 1 (chloroplastic)	It may participate in 4Fe-4S cofactor incorporation into psaA and/or psaB during translation	3e-140
5944	TSA *Abies pinsapo*	Ribonuclease H	DNA binding<br>Transferase activity	2e-67
